# Overwhelming Post-splenectomy Infection Caused by Escherichia coli 20 Years After Splenectomy: A Case Report

**DOI:** 10.7759/cureus.42184

**Published:** 2023-07-20

**Authors:** Yoshinobu Abe, Hideya Itagaki, Tomoyuki Endo

**Affiliations:** 1 Division of Emergency and Disaster Medicine, Tohoku Medical and Pharmaceutical University, Sendai, JPN

**Keywords:** escherichia coli, septic shock, overwhelming post-splenectomy infections, splenectomy, purpura fulminans

## Abstract

Post-splenectomy patients are at increased risk of infection. This complication is called overwhelming post-splenectomy infection (OPSI), which is uncommon but has high mortality. We describe a case of a man in his 80s who presented with septic shock with purpura fulminans caused by pyelonephritis. He had undergone a splenectomy in his 50s and had been taking prednisolone for the past six months for suspected immunoglobulin G4 (IgG4)-related disease. He was admitted to the intensive care unit but died the day after admission. OPSI is generally caused by encapsulated bacteria. However, in the present case, the causative agent was *Escherichia coli*, a bacterium that typically causes urinary tract infections. Post-splenectomy patients are known to have compromised bacterial clearance, and accumulation of bacteria such as *E. coli* can induce acute sepsis after splenectomy. Thus, physicians must have a high index of suspicion when treating splenectomy patients for the possibility that they may rapidly deteriorate to severe conditions such as OPSI, and the patients must be informed about the risk of severe infections, which can be fatal.

## Introduction

The pentameric immunoglobulin M (IgM) produced by IgM memory B-lymphocytes contributes to the elimination of encapsulated organisms, such as *Streptococcus pneumoniae*, *Neisseria meningitidis*, and *Haemophilus influenzae* [1]. IgM memory B-lymphocytes are a unique B cell population in the spleen. Thus, post-splenectomy patients have greatly reduced B-lymphocyte levels and are therefore prone to severe infection with encapsulated bacterial pathogens [1]. This complication is called overwhelming post-splenectomy infection (OPSI), which although rare, is associated with high mortality. We encountered a case of septic shock with purpura fulminans, associated with pyelonephritis, caused by *Escherichia coli* in a post-splenectomy patient who died the day after being admitted to the intensive care unit (ICU). The patient had apparently undergone a splenectomy when he was operated upon for pancreatic cancer over 20 years ago. The course of this case suggests that even though a post-splenectomy patient may not develop OPSI within a few years of splenectomy, they may do so many years later when they are at high risk for infections due to advancing age compounded with other risk factors such as diabetes and use of immunosuppressant drugs. This case report promotes awareness among physicians involved in post-splenectomy patients.

## Case presentation

A man in his 80s was brought to the emergency department of our hospital with complaints of altered consciousness. He had been visiting the rheumatology department of our hospital for suspected IgG4-related disease for eight months and was taking prednisolone orally for the previous six months. Initially, the patient was prescribed prednisolone 25 mg/day for 14 days. Thereafter, the dosage was tapered gradually to 17.5 mg/day; 22.5 mg/day for 35 days, 20 mg/day for 84 days, and 17.5 mg/day for 45 days. In addition, he had undergone surgery for bladder cancer and left renal pelvis cancer in his 80s. He had received no specific therapy for these cancers for the past four years. Additionally, he had diabetes and was taking oral antidiabetic medications (empagliflozin 10 mg/day, alogliptin 25 mg/day, and pioglitazone 15 mg/day) and subcutaneous insulin injections (insulin lispro 10-8-8 units and glargine 10 units/day). His hemoglobin A1c within one month was 6.2 %

The patient had visited the rheumatology department two days prior to the emergency transport to our hospital, and his physical condition was the same as his previous visit. Blood tests revealed a white blood cell (WBC) count of 8,000/μL and a C-reactive protein (CRP) level of 0.24 mg/dL, suggesting the absence of inflammatory reaction (Table 1). Urinalysis revealed no evidence of urinary tract infection. However, he complained of pain in his lower extremities during the night and had visited a surgical clinic for the pain on the day before he was transported to our hospital. The physician noted color changes in his lower extremities, and the patient was referred to an internal medicine clinic. On the same day, he visited his physician for diabetes. His temperature was 38.1°C. Cellulitis of the lower extremities was suspected, and he was advised to consult a dermatologist in our hospital the next day.

**Table 1:** Blood tests two days prior to the emergency transport to our hospital

Test name	Finding	Reference range
C-reactive Protein (mg/dL)	0.24	0.00-0.14
White Blood Cell Count (/µL)	8,000	3,300-8,600

On arrival at the emergency department of our hospital, physical examination showed the following: his Glasgow Coma Scale was E3V3M6; blood pressure, 85/42 mmHg; pulse, 111 beats/min; temperature, 40.3 °C; respiratory rate, 30 breaths/minute; and oxygen saturation, 98% on a face mask at 6 L/min. Symmetrical purpura was observed bilaterally on the lower legs, and exudate fluid was observed from a wound of the lower left leg.

Blood tests revealed the following: lactate, 2.1 mmol/L; urea nitrogen, 40 mg/dL; creatinine, 2.16 mg/dL; creatine kinase, 1076 U/L; creatine kinase myocardial band (MB), 23 U/L; troponin T, 2.450 ng/mL; CRP, 23.2 mg/dL; procalcitonin, 27.4 ng/mL; WBC count, 600/µL; platelet count, 116,000/µL; D-dimer, 17.98 µg/mL; and blood glucose, 32 mg/dL (Table 2). Urinalysis revealed nitrite and leukocyte esterase positivity. The WBC count in the urine sediment was ≥100/high-power field. Plain computed tomography (CT) of his cervical and pelvic region revealed hydronephrosis of the right kidney, with inflammation of the fat tissue around the right kidney. The CT scan revealed the absence of the spleen. We estimated that the patient had undergone a splenectomy because he was operated on for pancreatic cancer in his 50s according to the information provided by his family. Point-of-care transthoracic echocardiography performed to identify the cause of shock revealed decreased left ventricular anterior wall motion. Based on these findings, we diagnosed the patient with septic shock due to pyelonephritis.

**Table 2:** Blood tests at the time of presentation at the emergency department

Test name	Finding	Reference range
Total Bilirubin (mg/dL)	0.74	0.40-1.50
Urea Nitrogen (mg/dL)	40	8-20
Creatinine (mg/dL)	2.16	0.65-1.07
Creatine Kinase (U/L)	1,076	59-248
Creatine Kinase MB (U/L)	23	0-12
Troponin T (ng/mL)	2.450	0.000-0.099
C-reactive Protein (mg/dL)	23.20	0.00-0.14
Procalcitonin (ng/mL)	27.40	0.00-0.05
White Blood Cell Count (/µL)	600	3,300-8,600
Platelet (/µL)	116,000	158,000-348,000
D-Dimer (µg/mL)	17.98	0.00-0.10
Blood Glucose (mg/dL)	31	73-109
Lactate (mmol/L)	2.1	0.5-1.6

**Figure 1:**
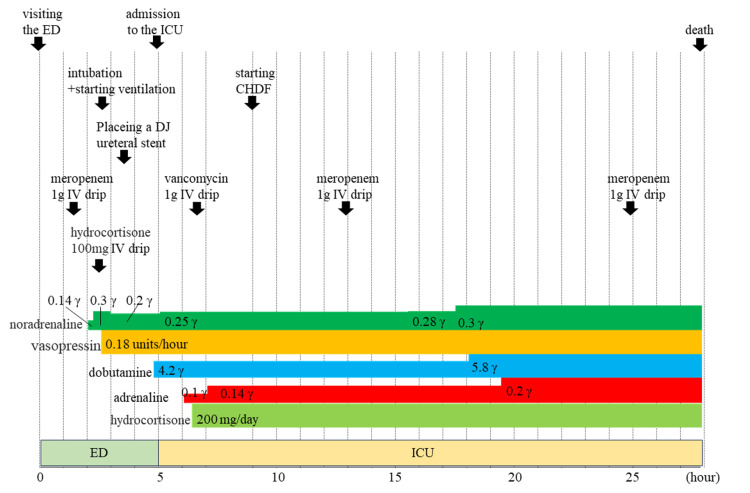
The timeline of treatment and dosing of drugs administered ED; Emergency department, ICU; Intensive care unit, CHDF; continuous hemodiafiltration, DJ ureteral stent; double-J ureteral stent, IV drip; intravenous drip, γ; µg/kg/minute

Three doses of 40 mL 50% glucose were administered for managing hypoglycemia. Vasopressors noradrenaline and vasopressin were administered for septic shock, along with infusions of intravenous fluids. However, these treatments did not increase the blood pressure; hydrocortisone was concurrently administered. Furthermore, since echocardiography showed decreased left ventricular systolic dysfunction, dobutamine was also administered. The patient's state of consciousness did not improve after performing resuscitation for circulation. Therefore, tracheal intubation was performed and mechanical ventilation was initiated in the emergency department. For treatment of pyelonephritis with septic shock, empirical therapy with the broad-spectrum antibacterial agent, meropenem, was initiated with an intravenous infusion at 2 g/day, and a double-J ureteral stent was placed for hydronephrosis (Figure 1).

His sequential organ failure assessment score at the time of ICU admission was 11 points, and he had multi-organ dysfunction syndrome (MODS). After admission to the ICU, continuous hemodiafiltration was initiated due to renal dysfunction and low urine output. As we could not maintain circulation, adrenaline was administered in addition to the aforementioned inotropes and vasopressors. Vancomycin (1 g) was also administered as an empirical therapy to further broaden the spectrum. However, the patient died of MODS due to septic shock the next day (Figure 1). 

**Figure 2:**
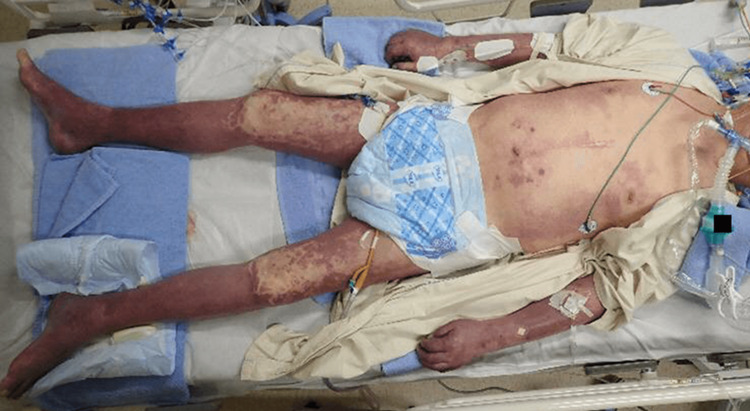
Purpura all over the body on the day after admission to the intensive care unit Symmetrical purpura was seen on both lower extremities as well as on face, both upper extremities, and the trunk. Face is not shown to avoid identification.

The patient developed purpura fulminans all over his body (Figure 2). *E. coli* was detected in two sets of blood culture (3+), the urine culture (3+), and the culture of exudate fluid from the left lower leg (1+). The bacterial volume in the urine culture was 10^6^ colony-forming units/ml. The results of the microbial susceptibility test were not suggestive of antimicrobial-resistant bacteria, such as extended-spectrum β-lactamase- and Amp-C β-lactamase-producing bacteria. The polymerase chain reaction was negative for the K1 antigen in this strain. There was no description of splenectomy, from his physician for diabetes, in the patient referral document to the dermatology department. In addition, no medical history of splenectomy was mentioned in the patient’s referral document from the previous hospital to our rheumatology department.

## Discussion

We presented a case of septic shock with purpura fulminans caused by *E. coli* in a post-splenectomy patient that was associated with a rapid disease course and death. The patient had undergone surgery for pancreatic cancer over 20 years prior, and it was believed that his spleen had been removed at that time. He had been taking prednisolone orally for six months for suspected IgG4-related disease. Furthermore, he had diabetes and was elderly.

In post-splenectomy patients, the highest risk of infections is within three years of splenectomy, but a high risk persists throughout life [2]. As in this case, several years following splenectomy, a patient may be assigned to a physician who may not be aware of the patient’s history of splenectomy. Therefore, physicians must educate the patients and their families about the high risk of severe infections in such cases, as patients may develop comorbidities in the years following splenectomy. The patient in the present case began taking steroids six months ago for suspected IgG4-related disease, which might have further exacerbated the risk of infections. Therefore, the steroid intake might also have contributed to the OPSI, which developed over 20 years after splenectomy. Additionally, his advanced age and diabetes were also relevant to this event.

*E. coli *is commonly found in the gastrointestinal tract and typically causes urinary tract infections [3]. OPSI occurs because the absence of the spleen compromises the capacity for the elimination of encapsulated organisms such as *S. pneumoniae*, *N. meningitidis*, and *H. influenzae*. However, based on our experience of the present case, we hypothesized that splenectomy might also affect the elimination of *E. coli*, so we searched for previous such studies. In an animal study, using rabbits intravenously injected with *E. coli*, it was reported that the clearance of *E. coli* in the blood was decreased in the splenectomized and splenic autotransplant groups compared to that in the control group [4]. The results of this study indicated that the spleen may play role in the elimination of *E. coli*. Thomsen et al. reported an increased risk of nosocomial infections in post-splenectomy cases [5]. They also reported that bacteremia caused by capsulated organisms such as *S. pneumoniae*, *N. meningitidis*, and *H. influenzae* was rare in the splenectomized group; and, *E. coli* was also a cause of bacteremia [5]. The risk of infection by E. coli may increase in post-splenectomy patients. In adults, prevention against *S. pneumoniae* and *N. meningitidis* by immunization is an option [6]. However, currently, there is no commercially available vaccine for *E. coli*, and there are no recommendations for it in the *Epidemiology and Prevention of Vaccine-Preventable Diseases* by the Centers for Disease Control and Prevention [6]. Therefore, if post-splenectomy patients are affected with any infectious disease, they must immediately visit a healthcare facility. Physicians must consider the possibility of such patients’ conditions becoming severe even if their symptoms appear mild.

## Conclusions

Based on the course of this case and a review of the literature, high-risk conditions for infections persist throughout life in post-splenectomy patients. Furthermore, immune response to *E. coli *is reduced in post-splenectomy patients. Even if the causative organism is commensal bacteria such as *E. coli*, which commonly causes urinary tract infections, the attending physician must consider the possibility that the condition of these patients may rapidly become severe such as OPSI. In addition, physicians must educate the patients and their families to be aware that they are continually at high risk for severe infections. Even if post-splenectomy patients do not develop severe infections within a few years after splenectomy, they remain at risk, which increases as they grow older and acquire additional risk factors for infections, such as diabetes and the use of immunosuppressive medications.
